# Biotechnological Approaches for Generating Zinc-Enriched Crops to Combat Malnutrition

**DOI:** 10.3390/nu11020253

**Published:** 2019-01-23

**Authors:** Kathleen Hefferon

**Affiliations:** Department of Food Sciences, Cornell University, Ithaca 14853 NY, USA; klh22@cornell.edu

**Keywords:** zinc deficiency (ZnD), biofortification, micronutrient, transgenic plant, soil, malnutrition

## Abstract

The past twenty years have seen the application of biotechnology to generate nutritionally improved food crops. Biofortified rice, cassava, maize, sorghum and other staple crops biofortified with essential micronutrients have great potential to benefit the world’s poor, in terms of both health and economics. This paper describes the use of genetic modification to generate crops that are biofortified with zinc. Examples of zinc-enhanced crops which have been developed using biotechnological approaches will be discussed, and new approaches for research and development will be outlined. The impact of these biofortified crops on human health and well-being will be examined. This paper will conclude with a discussion of the obstacles that must be overcome to enable zinc-fortified crops to be accessible for the world’s malnourished.

## 1. Introduction

Zinc is an important micronutrient for the growth and development of plants and animals. Zinc deficiency (ZnD) was first discovered in the 1960s and today, approximately one-third of the world’s population is zinc deficient. Zinc deficiency may result from poor diet or poor soils. Zinc is transported into crop plants directly from the soil, either in the form of Zn^2+^ or bound to an organic acid. Zinc can thus accumulate in the roots of plants and then become translocated through the xylem to the shoots and leaves. In plants, zinc has many roles, including membrane function, photosynthesis, gene expression, protection against drought and pathogens as well as for the synthesis of hormones that are involved in plant growth and development. Plants which lack adequate amounts of zinc suffer stunted growth and exhibit chlorosis on their leaves. Zinc is found at high levels of accumulation in seeds, indicating its importance in fertility [1]. 

**Figure 1:**
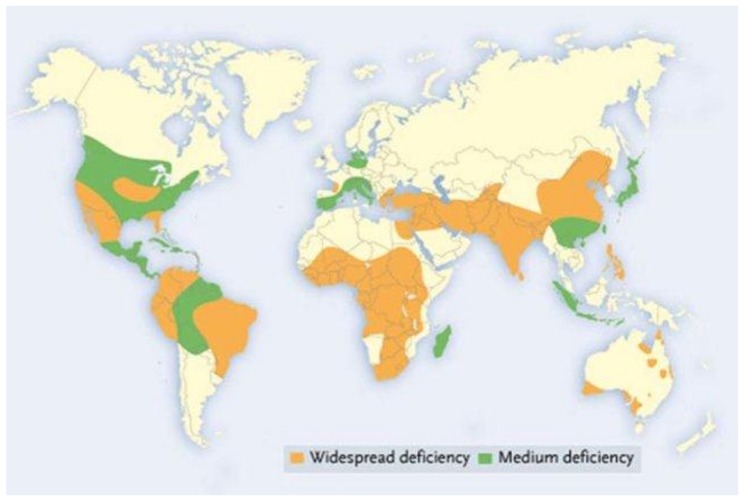
Geographical distribution of low Zn soil section in the world (adapted from Alloway, 2008). Alloway, B.J, (2004). Zinc in soils and crop nutrition. Brussels, Belgium: International Zinc Association [2].

Much of the world’s arable soils lack adequate levels of zinc (Figure 1). While soils that are alkaline and calcareous may be zinc-rich, it may not be taken up by plants under these conditions because zinc becomes tightly bound to the CaCO_3_ that is present in the soil. Zinc can also be found at levels that are toxic to life. For example, zinc toxicity in soils can arise from water sourced from irrigation, industrial runoff and soil erosion. 

More recently, climate change has been hypothesized to result in nutrient losses in crops [3,4]. A study performed on various crops exposed to higher carbon dioxide levels for prolonged time periods showed that rice, wheat and soybeans would accumulate less zinc. Worldwide, this could cause an additional 150–200 million people to fall into micronutrient deficiency than exist today [5,6].

Today, 17.3% of the world’s population is zinc deficient. Over 400,000 children under the age of 5 die every year due to zinc deficiency (ZnD). Zinc deficiency is responsible for growth and cognitive retardation, poor immune function as well as reproductive problems. Differences in zinc concentrations among different populaces can be due to poor concentrations in soils, differences in food preparation and processing practices among different cultures across the world, and even environmental pollution that concentrates in different geographical regions. The next section deals with the use of biotechnology to produce crops that are biofortified with zinc. Zinc-rich plants that have been developed using conventional plant breeding approaches (i.e., nontransgenic) will not be covered in this chapter. 

The following chapter describes methods of zinc biofortification as well as the importance of this micronutrient in human health. The chapter describes research that has been conducted into the molecular mechanisms behind zinc uptake into plants and possible use of this knowledge for biotechnological applications to improve zinc bioavailability. The chapter summarizes the genetic modification of food crops to increase zinc accumulation, using rice (Oryza sativa) as an example. The chapter concludes with an evaluation of the importance of future research on this topic in the years to come. 

## 2. Methods of Biofortification

Biofortification of plants can include both the breeding of new varieties of crops that are better able to take up micronutrients such as zinc, coupled with the external application of micronutrients in the form of fertilizers and foliar sprays [7]. Coating the seed with micronutrients such as iron and zinc prior to planting is another means by which to improve plant growth and development, as well as biofortifying plants to improve nutrition for human consumption. Although the use of externally added micronutrients in the form of fertilizers can be effective, the relative efficiency of biofortification can vary from one plant species to another. However, agronomic inputs such as fertilizers may not be affordable or accessible to the world’s rural poor. As a result, the use of new breeding techniques to create crop varieties which harbor higher levels of micronutrients such as iron and zinc are desirable [8,9].

### 2.1. Zinc and Human Biology 

#### 2.1.1. Importance of Zinc in Human Health

Zinc plays an essential role in the immune response [10]. For example, zinc ions are involved in regulating intracellular signaling pathways in immune cells, including B cells and T cells. Zinc homeostasis is controlled by zinc import proteins (ZIP) and zinc export proteins (ZNT) as well as other zinc binding proteins. Alterations in zinc homeostasis may result in disease development. For example, signs arising from zinc-deficiency that have been reported in animal studies include growth failure, hair loss, testicular atrophy and thickening and hyperkeratinization of the epidermis. 

Today, zinc deficiency (ZnD) is one of the leading sources of malnutrition. In developing countries, zinc deficiency is within the top five leading causes for the loss of healthy life years [11]. In industrialized countries such as the United States, zinc deficiency mostly affects the elderly, with nearly 30% of this population identified as being zinc deficient. This fact may contribute to chronic diseases which are associated with old age [10]. Besides iron, zinc is the second most abundant metal found in humans. While prostate, pancreas and bone are high in zinc, heart, brain and plasma are comparatively low in zinc [12]. Zinc is absorbed into the intestinal tract via zinc transporting proteins and is distributed throughout the body in a form that is bound to carrier proteins such as transferrin. Within the cytoplasm of living cells, zinc is bound by zinc-chelating proteins called metallothioneins (MTs) which function as zinc buffers. MTs are thus essential to zinc homeostasis, by keeping zinc below levels that are toxic to the cell but high enough to optimize cellular function [7].

#### 2.1.2. Zinc Homeostasis in the Human Digestive System

Zinc homeostasis is maintained in the human digestive system through the absorption of exogenous zinc and the exertion of endogenous zinc. Zinc uptake and transport into the bloodstream, where it can be circulated throughout the body, is essential for homeostasis. How zinc is taken up by cells is still not well understood. Subcellular uptake requires zinc transporter proteins, metallothionein (MT) and an intracellular metal binding protein, as well as other carrier proteins [13]. 

The efficiency of zinc absorption changes dramatically in both animal and human studies in response to changes in zinc availability. Absorption studies in animal models indicate an inverse relationship between percentage absorption and dietary zinc intake. Thus, zinc homeostasis can change depending on zinc availability and the physiological requirements of an individual. Unsurprisingly, milder forms of zinc deficiency are more likely to be widespread in certain vulnerable groups, including those with high physiologic requirements such as infants and young children, pregnant and lactating women and individuals chronically on low zinc intakes or diets with poor zinc bioavailability. For further information regarding zinc homeostasis in humans, please refer to the review article by Krebs [14]. 

### 2.2. Zinc and Plant Biology

#### 2.2.1. Mechanism of Zinc Uptake in Plants and Possibilities for Biotechnological Applications. 

**Figure 2:**
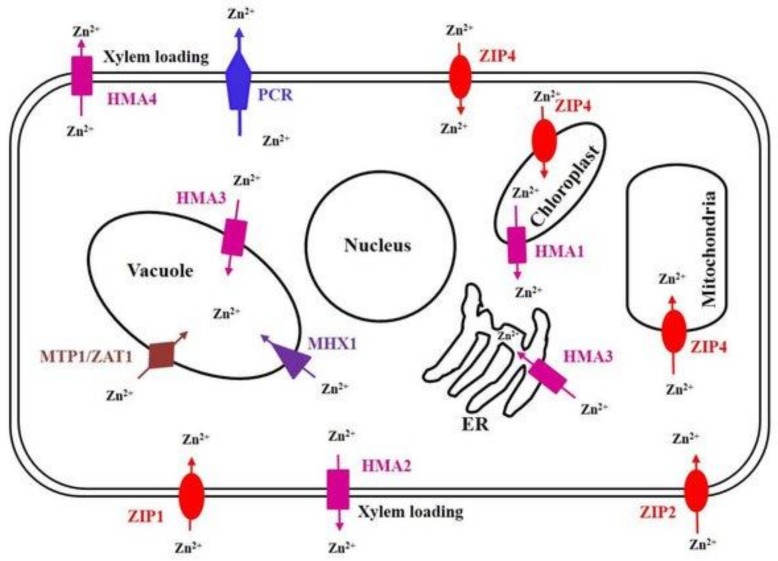
Localization of various Zn transporters in a plant cell. The Zn transporter family is actively involved in the uptake, transport, detoxification and homeostasis of Zn within plants. Depending on the Zn concentration in soil, various types of Zn transporters are expressed. During deficient concentration of Zn, ZIP (ZIP1, ZIP2 and ZIP4) and P-Type ATPase (HMA2), families of Zn transporters are induced which transport Zn into the cell through the plasma membrane from the soil, and then CAX (MHX1), CDF (MTP1 and ZAT1), P-Type ATPase (HMA2 and HMA4) and ZIP (ZIP4) families of transporters are involved in the mobilization of Zn into organelles. The PCR family member PCR2 is important for redistribution and detoxification Zn. The P-Type ATPase family member HMA1 is involved in detoxification of Zn in chloroplast. Studies on the localization and transport activity of ZIP transporters are still under progress [16].

**Figure 3:**
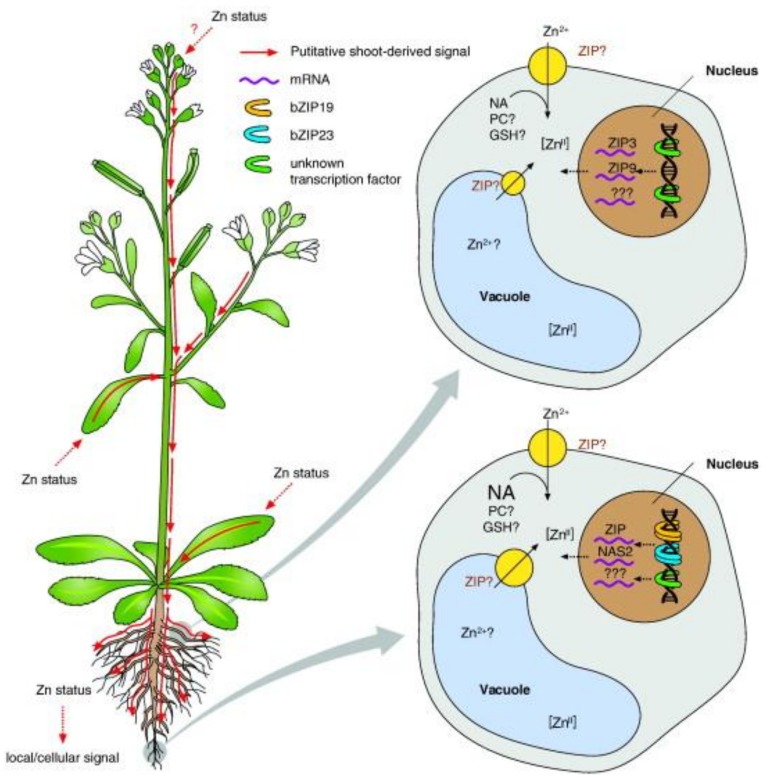
Potential signaling of Zn deficiency. Shown is a hypothetical model of proposed systemic (upper left and upper right) and local (lower left and lower right) responses to Zn deficiency in root cells. Increases in *ZIP3* and *ZIP9* transcript levels were observed in roots when *NgMTP1* was overexpressed in shoots (hypothesized to generate shoot-specific physiological Zn deficiency). This supports the existence of a putative systemic Zn-deficiency signal originating from the shoot that triggers Zn deficiency response gene expression in the root through unknown regulators. Local Zn deficiency results in the activation of *bZIP19* and *bZIP23*, which activate the transcription of a number of *ZIP* and *NAS* genes. Additional regulators may mediate further components of the Zn deficiency response. See main manuscript text for a detailed discussion [17].

Developing a better understanding of how Zn homeostasis is regulated in plants is of primary interest to biofortification and phytoremediation (the use of plants to remove contaminants) programs (Figure 2). Many reviews have been published on this subject, particularly regarding how zinc is loaded into the root zylem (Figure 3). Olsen and Palmgren [15] have demonstrated that zinc leaves the soil and is transported from symplast (an interconnected network of cells) to symplast through the use of apoplastic spaces. Zinc must also be passed across cell membranes, and a number of zinc transporter proteins have been identified. The ligand nicotianamine can bind to zinc and increase its solubility in living cells. Nicotianamine is a metal chelating molecule found at various levels in different plants, such as cereal crops, and reduces the bioavailability of micronutrients such as iron and zinc. All of these factors combined enables the intracellular zinc concentration to be finely regulated.

Johnson-Beebout et al. [18] examined soil Zn availability combined with zinc uptake in rice. Zn uptake pathways differed significantly by rice genotype (high-grain Zn varieties IR69428 and IR68144). The authors found that while variety IR69428 displayed high levels of zinc in stems, leaves and grain when planted in high zinc soil, variety IR68144 displayed increased zinc only in leaves during active tillering (shoot development). Since variety IR69428 displayed the highest levels of zinc accumulation, the authors proposed that the Zn uptake behavior of this rice genotype can determine how zinc travels from soil to grain, and thus can impact how zinc biofortification targets can be achieved for different rice varieties. Determining what differs among rice varieties and using this knowledge to produce a transgenic crop will be the next step needed to create zinc biofortified crops. 

Subbaiah et al. [19] used different concentrations of zinc oxide nanoparticulates (average size 25 nm) to determine their effect on the biofortification of maize crops compared to application with ZnSO_4_. Increase in yield, seed vigor and zinc content demonstrated that ZnO-nanoparticulates could play an important role in improving human health.

Yoneyama et al. [20] investigated the mechanisms and routes by which rice plants take up heavy metals such as iron and zinc. This was performed by determining the routes of metal transport and accumulation in rice plants by examining metal speciation, metal transporters, and the xylem-to-phloem transport system. When rice grain is undergoing storage, Zn that was ascending in the xylem sap is transferred to the phloem by the xylem-to-phloem transport system that operates at the location of stem nodes, by a process that is highly regulated. The authors suggested that biotechnological techniques that could increase metal chelator concentrations could increase grain Zn concentrations. 

#### 2.2.2. Lowering the Presence of Anti-Nutrients to Improve Zinc Bioavailability in Crops

Inositol hexaphosphates and pentaphosphates are phytic acids that act as anti-nutrients. Plant-produced phytic acids can bind to zinc and form insoluble complexes that result in reduced zinc absorption in the human digestive tract. Phytate is found to vary greatly among plant products, with grains (such as rice) and legumes (such as beans) having particularly high levels. The greater the amount of phytate present in a particular food crop, the less zinc accumulation takes place in the digestive tract. Besides poor soils, plant-based diets focusing on a single staple grain such as rice, with high phytate-to-zinc molar ratios, are a major factor contributing to zinc deficiency worldwide.

Genetic engineering to reduce inhibitors of zinc absorption such as plant phytates is thus another means by which to address worldwide zinc deficiency [21]. Reducing the level of phytates using RNAi technology, for example, would increase the bioavailability of zinc and other trace metal elements, thus increasing the nutritional benefits of many crop species [22]. RNAi technology involves the construction of an antisense RNA to a particular gene which can then block the expression of that gene. The antisense RNA would be generated in a transgenic plant. 

Reduction of phytic acid levels in cereal grains is desirable in view of its anti-nutrient properties, so as to maximize mineral bioavailability. Using RNA silencing in transgenic plants to lower phytate levels has led to an increase in the iron and zinc concentration in milled rice seeds [23,24]. Furthermore, Sakai et al. [25] demonstrated that decreased phytic acid in rice can lead to the accumulation and distribution of minerals within rice seed. 

#### 2.2.3. Outcome of Zinc Variability in Soil on Plants 

Zinc deficiency can be alleviated by increasing dietary Zn intakes through supplements or by Zn biofortification of edible crops [22]. Crops can undergo biofortification through the application of Zn-fertilizers in the soil, which are then taken up by the plant. Alternatively, crop varieties have been developed which acquire more Zn from the soil and then collect it in the edible portions. High concentrations of zinc can be achieved in roots and leaves with soil fertilizers and even with foliar Zn-fertilizers [26]. However, Zn concentrations in fruits, seeds, and tubers are generally significantly lower. Plants generated through biotechnology can translocate Zn through the phloem and thus increase Zn concentrations to the edible portions of plant tissues. 

Poblaciones and Rengel [27] applied zinc to soil and as a foliar spray to peas before flowering and at early grain-filling stage. Zinc concentrations increased 3.7- to 5.6-fold and grain zinc accumulation increased to 60 mg Zn kg(−1) with the foliar Zn applications, alone or in combination with soil Zn applications, suggesting that soil and foliar biofortification could work well for improving zinc bioavailability in field peas. However, processing bean grain (freezing and cooking) decreased Zn concentration by 30% and increased the phytate/Zn ratio by 17%. 

#### 2.2.4. How Plants Subsist on Zinc Deficient Soils 

Kappara et al. [28] identified two alleles of the *OsHMA7* transporter gene in rice by using *QTL* (quantitative trait locus) and analyzing lines which accumulated high and low levels of iron and zinc. The authors found that the overexpression of allele 284 in transgenic rice lines resulted in a high accumulation of iron and zinc, as well as tolerance to iron- and zinc-deficient soils. *OsHMA7* transcript levels were found to be five-fold higher in these transgenic lines. These changes in transcript levels also altered iron and zinc homeostasis in the plants by changing iron responsive gene expression. The authors concluded that they have identified a novel heavy metal transporter gene that can influence both grain yield as well as iron and zinc content, thus offering an improved line of rice for micronutrient-deficient populaces. 

Tiong et al. [29] measured zinc deficiency-induced uptake as well as root-to-shoot translocation in barley plants by examining the expression of the ZIP (ZRT/IRT-like protein) gene family. The authors resupplied 0.5 μM Zn to soil and quantified the transcripts of thirteen *HvZIP* genes. In the presence of Zn deficiency, both uptake and root-to-shoot translocation of Zn increased and this enhancement was sustained for days afterward. The authors found that six different *HvZIP* genes became induced in the roots of Zn-deficient plants; their corresponding proteins were localized to the plasma membrane of root cells. These ZIP genes can be analyzed further to identify how the plant is able to respond to fluctuating levels of zinc in the soil. Knowledge gathered from these studies will lead to new strategies for improving zinc accumulation in cereal crops, as well as provide tolerance for crops in zinc-deficient soils. 

#### 2.2.5. How Plants Subsist on Zinc Toxic Soils

The presence of iron and zinc ferrous iron (Fe) and zinc (Zn) at high concentrations in the soil can result in toxicity to crops such as rice and reduce both grain yield and quality. Plants have a natural mechanism of resistance against excess heavy metals such as iron and zinc. Using a genome-wide study, Zhang et al. [30] identified five toxicity tolerance QTL regions and 22 genes found within these regions will be further characterized for the mechanism of heavy metal tolerance. Knowledge gained from this research will help scientists to generate new crop varieties which are tolerant to high levels of metals such as iron and/or zinc.

### 2.3. India as a Case Study

Zinc deficiency is prevalent in rural populations where rice is the major source of calories in the diet. Stein et al. [31] estimated the burden of zinc deficiency (ZnD) in India expressed in disability-adjusted life years (DALYs) lost, for a survey that included 119,554 individuals. The study calculated that the burden of zinc deficiency is 2.8 million DALYs lost. Zinc biofortification of rice and wheat using plant breeding methods may thus reduce this burden by 20–51% and save 0.6–1.4 million DALYs each year. The cost for saving one DALY amounts to $US 0.73–7.31, thus biofortification through plant breeding would be extremely cost-effective by the standards of the World Bank and the World Health Organization. Similarly, Arsenault et al., 2010, found that zinc deficiency in rural Bangladesh among women and children is high (children (22%) and women (73–100%)). Increases in rice zinc content to levels that could be achieved through selective breeding could decrease zinc inadequacy to 9% in children and 20–85% in women. 

### 2.4. Biofortification Using Transgenic Plants 

Biofortified plants can also be developed using modern biotechnology. Transgenic crops that have been biofortified include rice, cassava, oilseeds, and potatoes. Micronutrients increased using biotechnology include iron, zinc, and vitamin A. Other nutritional factors such as essential amino acids and fatty acids, as well as reduced antinutritional factors (such as cyanogens and phytates) are also under development [12]. In the following section, transgenic rice will serve as an example of biofortification using genetic modification. An important goal of micronutrient biofortification is to enhance the amount of bioavailable zinc found in the edible seed of cereals. Since rice (oryza sativa) is an important grain to many of the world’s rural poor, substantial efforts have been made to create transgenic rice with improved zinc accumulation in the endosperm. Plant metal transporter proteins are able to use multiple metal substrates, including iron, zinc and even cadmium as substrates for uptake from soil into the roots. Mutations to create loss of function of these transporter proteins result in loss of uptake of all three of these metals into plant cells [32]. 

Masuda et al. [33] increased the accumulation of the iron storage protein ferritin as well as enhanced iron translocation by overexpressing the iron(II)-nicotianamine transporter OsYSL2 in rice endosperm. While yield remained similar to conventional rice, the transgenic lines produced higher levels of iron (6-fold in the greenhouse and 4.4-fold in the paddy) and zinc (1.6-fold). The authors concluded that the introduction of multiple genes involved in iron and zinc homeostasis is more effective for iron biofortification than the introduction of a single gene. Masuda et al. [34], successfully increased iron and zinc accumulation even more by enhancing the uptake and transport of iron using the ferric iron chelator, mugineic acid. In this case, the authors generated transgenic plants expressing the soybean ferritin gene (*SoyferH2*) driven by two endosperm-specific promoters, along with the barley nicotianamine synthase gene (*HvNAS1*), two nicotianamine aminotransferase genes (*HvNAAT*-A and -B), and a mugineic acid synthase gene (*IDS3*) to increase mugineic acid production in rice plants. Transgenic plants that were generated were shown to be tolerant to iron-deficient soil and increased iron concentrations 2.5-fold. Transgenic lines grown under iron-sufficient conditions increased iron accumulation by 4-fold as much as lines that were grown in both commercially supplied soil (iron-sufficient conditions) and calcareous soil (iron-deficient conditions). Lines expressing both ferritin and mugineic acid biosynthetic genes showed signs of iron-deficiency tolerance in calcareous soil. The iron concentration in polished T3 seeds was increased by 4- and 2.5-fold, as compared to that in non-transgenic lines grown in normal and calcareous soil, respectively. Thus, the authors were able to use ferritin and mugineic acid expressed in transgenic rice to increase accumulation in the seed, even under conditions that were iron-limited.

Addressing iron deficiency in rice can also result in an increased accumulation of zinc. For example, Aung et al. [35] generated a transgenic line of rice commonly used in Myanmar, where approximately 70% of the populace is iron deficient. These lines of transgenic rice overexpressed the nicotianamine synthase gene *HvNAS1* to enhance iron transport, the Fe(II)-nicotianamine transporter gene *OsYSL2* to transport iron to the endosperm and the Fe storage protein gene *SoyferH2* to increase iron accumulation in the endosperm. Transgenic rice plants that were generated accumulate more than 3.4-fold higher Fe concentrations, as well as 1.3-fold higher zinc concentrations compared to conventional, nontransformed rice, thus demonstrating that transgenic rice biofortified for increased iron content could address both iron and zinc micronutrient deficiency for the people of Myanmar. 

Rice tends to be low in iron and zinc due to the step of milling, which removes the nutrient-rich outer layers of the embryo. To mitigate this problem, Paul et al. [36] generated a transgenic high-yielding indica rice cultivar which expressed the ferritin gene from soybeans. These plants exhibited over a 2.6-fold higher level of ferritin, even in the fourth generation of rice plants. The plants, when milled, provided a 2.54-fold and 1.54-fold increase in iron and zinc content, respectively. Similarly, Tan et al. [37] used the iron transporter gene *MxIRT1* from apple trees to generate transgenic rice. Rice plants expressing *MxIRT1* exhibited a 3-fold increase in iron and zinc accumulation, yet a decrease in cadmium concentration. The authors concluded that MxIRT1 could act as an effective means to biofortify cereal crops such as rice. 

Trijatmiko et al. [38] also used a transgenic approach to demonstrate that plants which express rice nicotianamine synthase (*OsNAS2*) and soybean ferritin (*SferH-1*) genes showed an endosperm Fe and Zn enrichment. The authors used a Caco-2 cell assay to demonstrate that the increased iron and zinc is also bioavailable. 

Banakar et al. [39] developed transgenic rice plants that expressed nicotianamine and 2′-deoxymugenic acid (DMA) to accumulate iron and zinc in rice endosperm. Transgenic rice plants were capable of accumulating up to 4-fold more iron (Fe) and 2-fold more zinc (Zn) in rice endosperm, as well as lower levels of cadmium compared to wild-type plants, thus increasing the nutritional quality of rice plants for human consumption. Cadmium is thought to compete with iron and zinc for transport and accumulation in the rice endosperm. Lower amounts of cadmium result in a reduced toxicity in rice seed. 

## 3. Conclusions

Currently, approximately one-half of the world’s population is malnourished, lacking micronutrients such as zinc which are necessary to ensure human health [22]. Biofortification is a process by which micronutrients such as vitamins and minerals that are essential to global health can be supplied to food crops. While diet diversification and mineral supplementation are recognized approaches to relieve micronutrient deficiency, they are unavailable to many of the world’s rural poor. Biofortification can include agronomic applications, such as the use of fertilizers and foliar sprays, as well as the generation of new crop varieties with elevated levels of micronutrients through the use of new breeding technologies, such as genetic engineering. This paper has served to describe the state of plant biotechnology to address zinc deficiency across the world today. The health benefits of zinc, both in terms of crops and human health, have been discussed, as well as the results of zinc deficiency on living cells. The general mechanisms that are known for zinc uptake in plants have been described, as well as the soil conditions responsible for zinc deficiency or in such excess that toxic conditions are created. While much of the molecular architecture that concerns zinc homeostasis in plants and throughout crop species has not, as of yet, been clearly resolved, research studies that shed light on some of the key players that play important roles in the uptake of zinc from deficient soils are slowly undergoing identification and characterization. As a result, biotechnology can be applied using this increase in information so that new crop varieties which are resilient to zinc-deficient soils can be developed [40]. Recent research has focused on the overexpression of genes that are responsible for zinc uptake, transfer and accumulation in plant tissues, such as rice seed [21]. Biotech crops with reduced accumulation of anti-nutrients, such as phytic acid, are also under development through the use of RNAi technology [41]. The research described in this paper will thus increase zinc accumulation in staple crops as well as enable existing zinc stores within crops to become more bioavailable for the human diet. The advent of climate change and predicted changes in micronutrient accumulation in the world’s most important crops will make this research even more valuable in the years to come.
